# Losartan therapy in adults with Marfan syndrome: study protocol of the multi-center randomized controlled COMPARE trial

**DOI:** 10.1186/1745-6215-11-3

**Published:** 2010-01-12

**Authors:** Teodora Radonic, Piet de Witte, Marieke JH Baars, Aeilko H Zwinderman, Barbara JM Mulder, Maarten Groenink, COMPARE study group

**Affiliations:** 1 Department of Clinical Epidemiology, Biostatistics and Bioinformatics, Academic Medical Center Amsterdam, the Netherlands; 2 Department of Cardiology, Academic Medical Center Amsterdam, the Netherlands; 3 Department of Clinical Genetics, Academic Medical Center Amsterdam, the Netherlands; 4 Department of Radiology, Academic Medical Center Amsterdam, the Netherlands

## Abstract

**Background:**

Marfan syndrome (MFS) is one of the most common systemic disorders of connective tissue with the incidence of approximately 2-3 per 10 000 individuals. Aortic disease, leading to progressive aneurysmal dilatation and dissection is the main cause of morbidity and mortality of Marfan patients. Current treatment (e.g. beta blockers and elective surgery) does postpone but cannot prevent aortic complications in these patients. Recent studies have found Transforming Growth Factor β (TGF β) to be involved in the aortic aneurysm formation. Losartan, an Angiotensin II type 1 receptor blocker inhibits TGFβ in a mouse model of Marfan syndrome leading to inhibition of aortic growth. The main objective of this trial is to assess whether losartan treatment leads to a clinically relevant decrease of aortic dilatation in adult patients with Marfan syndrome.

**Methods/Design:**

COMPARE study (COzaar in Marfan Patients Reduces aortic Enlargement) is an open-label, randomized, controlled trial with blinded end-points. Treatment with losartan will be compared with no additional treatment after 3 years of follow-up. We will enroll 330 patients with MFS who will be randomly assigned to receive losartan or not. Patients taking beta-blockers will continue taking their standard treatment. The primary end-point is the largest change in aortic diameter at any aortic level measured by means of MRI. Secondary end-points are change in mortality, incidence of dissection, elective aortic surgery, aortic volume, aortic stiffness and ventricular function. We will also investigate gene and protein expression change in the skin under losartan therapy and create prediction models for losartan-treatment response and aortic dilatation.

**Discussion:**

The COMPARE study will provide important evidence of effects of losartan treatment in adult Marfan patient population. We expect losartan to significantly reduce the occurrence and progression of aortic dilatation. This trial investigates a wide spectrum of clinical, genetic and biochemical effects of losartan aiming to provide further insight in the pathogenesis and treatment of Marfan syndrome.

**Trial registration:**

Netherlands Trial Register NTR1423.

## Background

Marfan syndrome (MFS) is one of the most common systemic disorders of connective tissue with the incidence of approximately 2-3 per 10 000 individuals [1]. MFS is an autosomal dominant disorder with cardinal clinical features involving the cardiovascular, ocular and skeletal systems. MFS is a clinical diagnosis which is made when an index patient fulfils major criteria in two systems and has an involvement in a third system according to Ghent criteria[2]. Clinical features involve ocular lens luxation, long bone overgrowth, lung emphysema and cardiovascular complications including aortic aneurysm and mitral valve prolaps.

Aortic disease, leading to progressive aneurysmal dilatation and dissection is the main cause of morbidity and mortality of Marfan patients. Routine monitoring of aortic growth is necessary to detect and quantify the progression of aortic dilatation, in combination with β adrenergic receptor antagonist therapy to slow down aortic growth[3]. Prophylactic aortic repair is indicated when dilatation reaches sufficient size to threaten aortic dissection[4]. Current therapy has improved life expectancy of this group of patients. However, it is important to note that even treated patients continue to have abnormal aortic growth. Moreover, dissection may occur in not-operated parts of the aorta. Therefore, current treatment does postpone but cannot prevent aortic complications in these patients.

MFS is caused by mutations in FBN1, the gene encoding fibrillin-1, which is the main component of the extracellular matrix[5]. Fibrillin-1 was initially thought to play primarily a structural role in connective tissue. Some of the features of Marfan syndrome like lens luxation can be explained by weakness of connective tissue due to a defect fibrilline-1. However, this hypothesis was insufficient to clarify the pathogenesis of other features, like bone overgrowth and facial appearance in these patients. The additional role of fibrilline-1 as a regulator of a cytokine named Transforming Growth Factor β (TGF-β) emerged [6-8]. TGF-β is a growth factor which is involved in many biological processes including cellular proliferation, deposition of the extracellular matrix, differentiation, apoptosis etc [6-9]. In a mouse model of MFS, increased TGF-β signaling appeared to play a causal role in many phenotypic features of MFS like progressive aortic root dilatation, defect lung development resulting in bullae formation and failed muscle regeneration [6,7,9,10].

These new insights in the pathophysiology of MFS are a major step forward in the understanding of connective tissue disorders, offering new opportunities for treatment of these patients.

Angiotensin II type 1 receptor (AT1) blocker losartan was found to be potentially useful in MFS because it leads to antagonism of TGF-β in animal models of chronic renal insufficiency and cardiomyopathy[11]. Numerous studies describe the ability of losartan to achieve a clinically relevant inhibition of TGFβ. This led to an assumption that losartan can treat or even prevent some features of MSF. Promising preliminary results on aortic dilatation inhibition have been achieved in a small observational study with children with severe MFS[10].

MFS is a very pleiotropic disorder. Up-till now no parameters of genotype-phenotype correlation have been found [12]. Neither the location of the mutation nor the type of aminoacid altered nor even the family presentation are sufficient to predict the phenotype both among as within the affected families[13]. These findings suggest that other genes may be involved in the pathogenesis modifying the phenotype.

## Methods/Design

**Figure 1:**
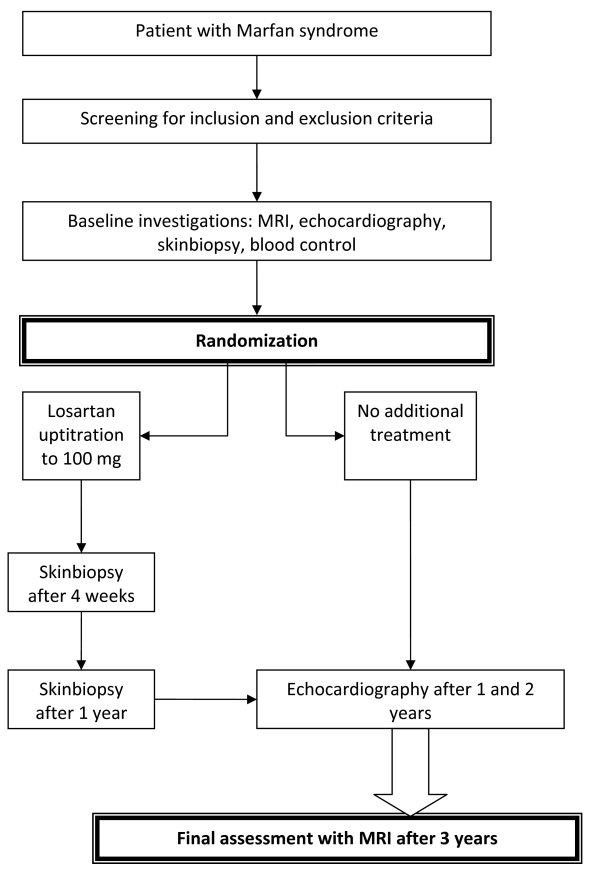
**Flow of participants, COMPARE study**.

The COMPARE trial is an open-label, randomized, controlled trial with blinded end-points. Treatment with losartan will be compared with no additional treatment after 3 years of follow-up. We will enroll 330 patients with MFS who will be randomly assigned to receive losartan or not. Patients taking beta-blockers will continue taking their standard treatment. This multi-center study will be conducted in four academic centers in the Netherlands: Academic Medical Center in Amsterdam, University Medical Center St. Radboud in Nijmegen, University Medical Center Groningen and Leiden University Medical Center. A flow chart of the study design is shown in the Figure 1.

### Objectives

#### Primary objective

The main objective of this study is to assess whether losartan reduces aortic dilatation at any aortic level, from the aortic root to the bifurcation. The primary end-point is the largest aortic diameter at any level measured by means of Magnetic Resonance Angiogram (MRA). If MRA is contraindicated, a Computed Tomography (CT) will be performed.

We will therefore test the null hypothesis that there is no significant difference in aortic diameters after 3 years of follow-up in the treated and the control groups. MRA's will be assessed by a person who is blinded for patient's treatment.

#### Secondary objectives

The secondary objectives are to asses whether losartan influences:

• Mortality, incidence of newly diagnosed dissection in any main vessel and incidence of elective aortic surgery combined into one event endpoint

• Aortic volume measured by means of MRA

• Aortic pulse wave velocity (PWV) and distensiblity measured by means of Magnetic Resonance Imaging (MRI)

• Ventricular function measured by means of MRI

#### Objectives of genetic expression studies

This study aims also to provide further understanding of the pathofysiology of MFS. Skin samples will be used as a model of the aortic tissue. We will assess gene expression in the skin samples of the treated patients at three points: at baseline, after four weeks of treatment and after one year of losartan treatment. Genome wide expression will be measured to identify genes the expression of which changes under losartan therapy. The expression analysis is mainly exploratory and will be performed also for pathways involved in the pathogenesis of Marfan syndrome, i.e. TGF-β pathway. Expression panels will be correlated with the rate of aortic dilatation. Results will be used to build prediction models of the losartan-therapy response and to identify genes which may influence the phenotype. Results will be validated in aortic tissue collected from Marfan patients operated in one of the four participating centers.

### Inclusion criteria are

- Diagnosis of MFS according to Ghent criteria

- Age ≥ 18 years

### Exclusion criteria

- More than one vascular prosthesis

- Aortic root diameter > 50 mm

- Aortic dissection

- Renal dysfunction (creatinine > 130 μg/ml and/or K > 5 mmol/ml)

- Treatment with Angiotensin-Converting Enzyme (ACE) inhibitors or Angiotensin Receptor Blockers (ARB's)

- History of angioedema or other intolerance to ACE-inhibitors and ARB's. 

- Intolerance of intravenous contrast for MRI/CT.

- Aortic surgery within 6 months of inclusion

Eligible patients will be randomly assigned in a 1:1 ratio to receive losartan 2 × 50 mg or not. We will start with 50 mg and double the dosage to 100 mg after 14 days. The maximal tolerable dosage will be continued. Randomization is stratified according to site, with blocks of 10.

### Follow-up

After inclusion the following will be obtained during the baseline investigations (V1):

• Patients medical history

• Echocardiography

• MRI or a CT of the heart and entire aorta

• Blood samples

• Punch skin biopsy

Patients will be evaluated after one, two and three years of treatment by means of echocardiography. After 4 weeks and one year of the treatment an additional skin biopsy will be obtained. At every visit, patient's medical history will be obtained. Date of dissection, operation or death will be carefully noted.

The final assessment will be after 3 years of treatment by means of MRI.

## Ethical aspects and trial organization

The protocol has been approved by the Medical Ethical Committee of the Academic Medical Center in Amsterdam. The feasibility approvals have been obtained of all the participating centers. This trial is registered in the Netherlands Trial Register under number NTR1423. Enrollment began in March 2008 and in October 2009 230 patients have been enrolled.

## Statistical considerations

Mean change in aortic diameter in patients with MFS is 0.9-1.5 mm/year[4]. Sample size calculation is based on the primary endpoint (rate of change of aortic diameter). The expected difference between two groups is 0.5 mm/year with a standard deviation of 1.5 mm (2-sided α = 0.05; β = 0.2). Based on these assumptions we calculated that 286 patients are required. To compensate for an estimated 20% drop-out, the inclusion of at least 330 patients is advised.

Primary analyses will be performed on an intention to treat basis. To evaluate the primary end-point a covariate analysis of the diameter change will be used in two groups (no treatment vs losartan) at baseline and after 3 years. Covariate analyses will be also used for the following secondary points: aortic volume, distensibility, pulse-wave velocity, left and right ventricle volumes. "Safety parameters" (i.e. mortality, dissections, surgery) will be evaluated by means of a X^2 ^test.

## Discussion

COMPARE study will provide important evidence of effects of losartan treatment in adult Marfan patients and lead to evidence-based recommendations. It is of immense importance for this trial to be conducted before Losartan (Cozaar^®^), an existent anti-hypertensive drug, is subscribed to Marfan patients based on promising preliminary results. This study aims to provide further insight in the pathogenesis and genetics of Marfan syndrome unraveling the phenotype-genotype correlation and may provide more evidence based support for the counseling of this group of patients. Insights in the causes and treatment of the aortic dilatation in MFS and therefore the integrity of vascular wall can undoubtedly be extended to other common types of aneurysm.

## List of abbreviations

MFS: Marfan syndrome; TGF-β: Transforming Growth Factor β; AT1: Angiotensin II type 1 receptor; MRA: Magnetic Resonance Angiogram; CT: Computed Tomography; PWV: Pulse wave velocity; MRI: Magnetic Resonance Imaging; ACE: Angiotensin-Converting Enzyme; ARB: Angiotensin Receptor Blockers.

## Competing interests

The authors declare that they have no competing interests.

## Authors' contributions

TR participated in design and execution of the trial and drafted this manuscript. PW participated in execution of the trial. MB contributed to the design of the trial. AZ participated in the design and methodological considerations of the trial. BM participated in the design and coordination of the trial. MG participated in conceiving and coordination of the trial.
